# Water, sanitation and hygiene interventions for acute childhood diarrhea: a systematic review to provide estimates for the Lives Saved Tool

**DOI:** 10.1186/s12889-017-4746-1

**Published:** 2017-11-07

**Authors:** Nazia Darvesh, Jai K. Das, Tyler Vaivada, Michelle F. Gaffey, Kumanan Rasanathan, Zulfiqar A. Bhutta, for the Social Determinants of Health Study Team, Zulfiqar A. Bhutta, Nazia Darvesh, Andreea Seusan, Jelena Savic, Nisso Nurova, Azim Rattansi, Daina Als, Tyler Vaivada, Michelle F. Gaffey, Sue Cavill, Kumananan Rasanathan, Jai K. Das

**Affiliations:** 1 Centre for Global Child Health, The Hospital for Sick Children, 686 Bay Street, Toronto, ON M6S 1S6 Canada; 2 Division of Women and Child Health, Aga Khan University, Karachi, Pakistan; 3 UNICEF, New York, NY USA; 4 Centre of Excellence in Women and Child Health, Aga Khan University, Karachi, Pakistan

**Keywords:** Lives saved tool, LiST, Water, Sanitation, Hygiene, Wash, Diarrhea

## Abstract

**Background:**

In the Sustainable Development Goals (SDGs) era, there is growing recognition of the responsibilities of non-health sectors in improving the health of children. Interventions to improve access to clean water, sanitation facilities, and hygiene behaviours (WASH) represent key opportunities to improve child health and well-being by preventing the spread of infectious diseases and improving nutritional status.

**Methods:**

We conducted a systematic review of studies evaluating the effects of WASH interventions on childhood diarrhea in children 0–5 years old. Searches were run up to September 2016. We screened the titles and abstracts of retrieved articles, followed by screening of the full-text reports of relevant studies. We abstracted study characteristics and quantitative data, and assessed study quality. Meta-analyses were performed for similar intervention and outcome pairs.

**Results:**

Pooled analyses showed diarrhea risk reductions from the following interventions: point-of-use water filtration (pooled risk ratio (RR): 0.47, 95% confidence interval (CI): 0.36–0.62), point-of-use water disinfection (pooled RR: 0.69, 95% CI: 0.60–0.79), and hygiene education with soap provision (pooled RR: 0.73, 95% CI: 0.57–0.94). Quality ratings were low or very low for most studies, and heterogeneity was high in pooled analyses. Improvements to the water supply and water disinfection at source did not show significant effects on diarrhea risk, nor did the one eligible study examining the effect of latrine construction.

**Conclusions:**

Various WASH interventions show diarrhea risk reductions between 27% and 53% in children 0–5 years old, depending on intervention type, providing ample evidence to support the scale-up of WASH in low and middle-income countries (LMICs). Due to the overall low quality of the evidence and high heterogeneity, further research is required to accurately estimate the magnitude of the effects of these interventions in different contexts.

## Background

Clean water, availability of toilets and good hygiene practices are essential for the survival and development of children. Globally, there are 2.4 billion people who live without adequate sanitation, 663 million do not have access to improved water sources and 946 million still defecate in the open [1]. While there has been progress, it has been slow and uneven, with 96% of the global urban population using improved drinking water sources in 2015 compared to 84% of the rural population; 82% of the global urban population uses improved sanitation facilities compared to 51% of the rural population [1].

Children under the age of five years are the most affected as they are prone to water-borne diseases, especially diarrhea. It is estimated that over 800,000 children die annually from preventable diseases caused by poor water, lack of sanitation and poor hygiene [2]. Diarrhea is one of the leading causes of morbidity and mortality in children, and while there has been progress in the reduction of diarrhea-associated mortality [3], the reduction in incidence and morbidity has varied in different regions and between socio-economic classes. In particular, the relationship of early exposure to pathogens, diarrheal burdens, and high rates of stunting, also called environmental enteropathy, is well appreciated [4]. Poor status of water, sanitation and hygiene (WASH) and related interventions can impact growth and development of children in multiple ways [4] and there is consensus that improvement in undernutrition would not be possible without improving WASH conditions of underprivileged children around the world.

There are several interventions for improving WASH that have been implemented in varying contexts worldwide, with the evidence evaluated for their impact on health and social outcomes. The evidence so far has been sparse, complex, and not of sufficient quality to propose any conclusive impact of these interventions on broader health and other outcomes. Some of these difficulties relate to endpoints such as environmental enteropathy or developmental outcomes, and in other instances studies are not sufficiently powered to assess mortality outcomes. Diarrhea is a relevant outcome that has been evaluated relatively rigorously and has been used extensively in previous reviews to evaluate the effectiveness of WASH interventions in childhood [3–10]. We aimed to update the evidence synthesis presented by Cairncross et al. [7] which has guided interventions for the existing Lives Saved Tool (LiST) since 2010, and to propose fresh estimates for modeling within LiST.

## Methods

### Search and data abstraction

We systematically reviewed the published literature up to September 2016. We relied on a search that was previously conducted by our team for a broader evaluation of WASH interventions in September 2014 and updated that search in September 2016 to incorporate relevant new evidence. The search was conducted in Medline, CINAHL, EMBASE, CAB Abstracts, Cochrane, BLDS, EconLit, IDEAS, SIGLE, WHOLIS and JOLIS. Further articles from secondary sources were retrieved by screening the reference list of a Gapmap by Waddington and colleagues [11] and the reference lists of relevant reviews and reports [3–9]. A search strategy was designed including Medical Subject Heading Terms (MeSH) and keywords using various combinations. No language or date restrictions were employed in the electronic searches.

We initially screened, in duplicate, the titles and abstracts of retrieved articles to determine whether they met our inclusion and exclusion criteria. The full-texts of all selected studies were then retrieved and assessed by two reviewers for eligibility. In duplicate, we abstracted descriptive and quantitative data from included studies into a standardized form.

### Inclusion/exclusion criteria

Two authors independently assessed study eligibility using pre-defined inclusion and exclusion criteria. Discrepancies between the reviewers in the decision to include or exclude studies were resolved by discussion aimed at reaching consensus or by consulting with a third author.

We limited included studies to randomized controlled trials (RCTs), cluster randomized controlled trials (cRCTs) and quasi-experimental (QE) trials where the following WASH interventions were evaluated in community settings in children 0–5 years old:

1. Water quality improvement at source and point-of-use
2. Promotion of handwashing with soap
3. Safe excreta disposal

We included studies published in English that evaluated the impact of these interventions on acute childhood diarrhea in children 0–5 years old. Our outcomes of interest included diarrhea-related mortality, diarrhea-related morbidity and risk of diarrhea. We excluded studies reporting only behavioral outcomes. We excluded studies comparing the effect of different interventions without a control group; studies conducted in specific settings such as schools, daycares, and hospitals; studies where the intervention was the use of hand scrubs or disinfectants; studies measuring the impact on dysentery only, specific pathogens such as cholera or soil-transmitted helminths (STHs), or general gastrointestinal outcomes like highly-credible gastrointestinal illness (HCGI); studies conducted in emergency settings or refugee camps; or studies conducted only with specific populations such as HIV-infected persons. We also excluded studies where multiple interventions were evaluated together and the impact of a single intervention could not be inferred, or where the data were not reported sufficiently to be included in a meta-analysis.

### Assessment of risk of bias

The quality of studies was assessed using methods adapted from the Cochrane ‘Risk of bias’ assessment tool [12] and the Child Health Epidemiology Reference Group (CHERG) guidelines [13]. For each study, two reviewers independently assessed the quality of included studies for the following domains; allocation concealment, sequence generation; blinding of outcome assessors, blinding of participants and personnel, and incomplete outcome data. During quality assessment, RCTs and cRCTs started at a ‘high’ rating and quasi-experimental (QE) studies started at a ‘low’ rating with each study’s rating adjusted accordingly and given either high, moderate, low or very low scores. Where a study reported multiple outcomes, we assigned a separate overall study score for each, depending on how the outcome was measured.

### Data analysis

We entered the abstracted effect estimates into Review Manager (RevMan) 5.3 and made calculations where necessary [12, 14]. In duplicate, the effect of the interventions on diarrheal outcomes was extracted, and calculated when necessary. These included risk ratios (RRs), odds ratios (ORs), rate ratios, means ratios, and longitudinal prevalence ratios, depending on how the individual study authors chose to display the effect. For treating all effect measures as equivalent, the design effect was considered for the various effect measures for common outcomes like diarrhea. The different measures of effect were then converted to a single measure for such outcomes [15]. In our analysis, ORs were transformed into RRs using an assumed control risk and formula recommended by Higgins et al. [12].

Where studies presented outcomes at different time points, we selected the effect estimate from the longest follow-up time. When studies provided effect estimates separated into different age strata of children 0–5 years old, we combined the point estimates from each stratum in RevMan using fixed effects models and then added the resulting pooled effect estimate into our main meta-analysis [16]. To quantitatively synthesize the available evidence, we grouped together similar intervention and outcome types and conducted meta-analyses using the generic inverse variance method. Random effects models were used to estimate the average effect of the intervention under the assumption that the intervention effects from individual studies were drawn from a distribution of effects rather than indicating the same fixed effect. For each intervention-outcome pair, the pooled RR was reported with a 95% confidence interval (CI). Subgroup analysis was conducted for the difference in the intervention.

### Quality of evidence

After each study was assessed for methodological quality and assigned a rating according to the CHERG adaptation of the GRADE technique [13], the quality of the overall evidence for each intervention and outcome combination was assessed on a four-level scale (high, moderate, low, very low).

## Results

**Fig. 1:**
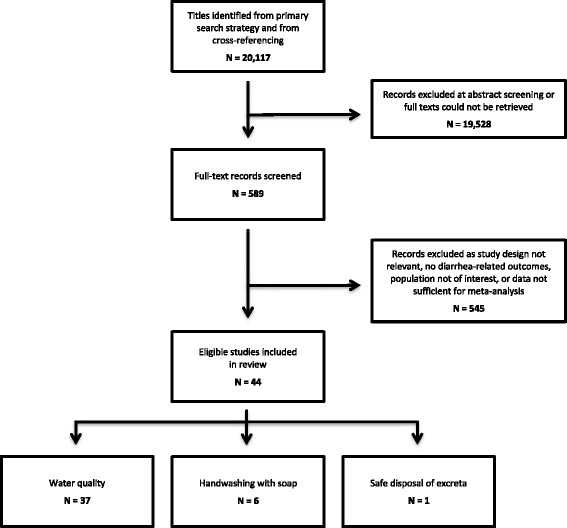
Search flow diagram

Figure 1 shows the results of the search strategy and altogether a total of 44 studies were identified to be included in the review. The characteristics of included studies are described in Table 1. The quality assessment of these studies suggests that the evidence is of low to very low quality (Table 2).

**Table 1:** Characteristics of included studies

Study	Country	Study design	Intervention	Estimates on diarrhea (RR [95% CI])
Improved water quality at source				
Alam 1989 [17]	Bangladesh	QE	Hand Pump	0.83 [0.71, 0.97]
Opryszko 2010 [18]	Afghanistan	cRCT	Hand Pump	1.22 [0.86, 1.73]
Jensen 2003 [19]	Pakistan	QE	Chlorination	0.95 [0.35, 2.60]
Ryder 1985 [20]	Panama	QE	Improved Supply	1.34 [1.11, 1.62]
Semenza 1998 [21]	Uzbekistan	cRCT	Improved Supply	0.65 [0.44, 0.95]
Improved water quality at point-of-use				
Water Filtration				
Aceituno 2012 [22]	Honduras	RCT	Biosand Filter	0.62 [0.36, 1.08]
Boisson 2009 [23]	Ethiopia	RCT	Lifestraw	0.97 [0.67, 1.40]
Boisson 2010 [24]	Democratic Republic of Congo	RCT	Lifestraw	0.85 [0.56, 1.29]
Brown 2007 [25]	Cambodia	QE	Ceramic Filter	0.52 [0.32, 0.85]
Brown 2008 [26]	Cambodia	RCT	Ceramic Filter (Iron rich)	0.58 [0.41, 0.82]
			Ceramic Filter with Vessel	0.65 [0.46, 0.92]
Clasen 2004 [27]	Bolivia	RCT	Ceramic Filter	0.41 [0.17, 1.02]
Clasen 2005 [28]	Colombia	RCT	Ceramic Filter	0.63 [0.45, 0.89]
Du Preez 2008 [29]	South Africa and Zimbabwe	RCT	Ceramic Filter	0.21 [0.12, 0.37]
Lindquist 2014 [30]	Bolivia	cRCT	Hollow water filter	0.21 [0.15, 0.29]
			Hollow water filter with behavior change campaign	0.27 [0.22, 0.33]
Stauber 2009 [31]	Dominican Republic	RCT	Biosand Filter	0.46 [0.35, 0.60]
Stauber 2012a [32]	Ghana	cRCT	Biosand Filter	0.26 [0.07, 0.97]
Stauber 2012b [33]	Cambodia	cRCT	Biosand Filter	0.45 [0.26, 0.78]
Tiwari 2009 [34]	Kenya	cRCT	Biosand Filter	0.49 [0.24, 1.00]
Water Disinfection				
Boisson 2013 [35]	India	RCT	Chlorination	0.95 [0.79, 1.14]
Chiller 2006 [36]	Republic of Guatemala	cRCT	Flocculent disinfectant	0.63 [0.48, 0.82]
Crump 2005 [37]	Kenya	cRCT	Flocculent disinfectant	0.75 [0.59, 0.95]
			Chlorination	0.83 [0.66, 1.04]
Du Preez 2011 [38]	Kenya	RCT	SODIS	0.73 [0.63, 0.85]
Harshfield 2012 [39]	Haiti	QE	Chlorination	0.61 [0.45, 0.83]
Jain 2010 [40]	Ghana	RCT	Chlorination	1.13 [0.92, 1.39]
Kirchhoff 1985 [41]	Brazil	QE	Chlorination	0.97 [0.84, 1.12]
Luby 2006 (1) [42]	Pakistan	cRCT	Chlorination	0.39 [0.17, 0.89]
			Flocculent disinfectant	0.54 [0.31, 0.94]
Mahfouz 1995 [43]	Saudi Arabia	QE	Chlorination	0.55 [0.30, 1.00]
McGuigan 2011 [44]	Cambodia	cRCT	SODIS	0.37 [0.29, 0.47]
Mengistie 2013 [45]	Ethiopia	RCT	Chlorination	0.43 [0.38, 0.49]
Mausezahl 2009 [46]	Bolivia	cRCT	SODIS	0.74 [0.50, 1.10]
Opryszko 2010 [18]	Afghanistan	cRCT	Chlorination	1.20 [0.84, 1.71]
Quick 1999 [47]	Bolivia	cRCT	Chlorination	0.56 [0.45, 0.69]
Rai 2010 [48]	India	RCT	SODIS	0.24 [0.10, 0.60]
Reller 2003 (1) [49]	Republic of Guatemala	RCT	Chlorination	0.77 [0.29, 2.08]
			Chlorination with vessel	0.92 [0.65, 1.30]
			Flocculent disinfectant	0.69 [0.50, 0.95]
			Flocculent disinfectant with vessel	1.05 [0.78, 1.41]
Rose 2006 [50]	India	QE	SODIS	0.64 [0.48, 0.86]
Semenza 1998 [21]	Uzbekistan	cRCT	Chlorination	0.33 [0.19, 0.57]
Sobsey 2003 [51]	Bangladesh	RCT	Chlorination	0.78 [0.73, 0.83]
HANDWASHING WITH SOAP				
Han 1989 [52]	Myanmar	cRCT	With Provision of Soap	0.70 [0.54, 0.93]
Langford 2011 [53]	Nepal	cRCT	With Provision of Soap	0.74 [0.54, 1.01]
Luby 2004a [54]	Pakistan	cRCT	With Provision of Soap	0.55 [0.45, 0.68]
Nicholson 2014 [55]	India	cRCT	With Provision of Soap	1.10 [0.77, 1.57]
Shahid 1996 [56]	Bangladesh	QE	With Provision of Soap	0.53 [0.44, 0.62]
Sircar 1987 [57]	India	QE	With Provision of Soap	1.13 [0.79, 1.62]
Safe disposal of excreta				
Clasen 2014 [64]	India	cRCT	Latrine promotion and construction	0.97 [0.83–1.12]

**Table 2:** Quality assessment of the evidence

	Quality Assessment				
Number of studies	Study design(s)	Limitations	Consistency	Generalizability	Overall quality of evidence (justification)
Effect Of Water Quality Interventions at Source					
*Outcome: Diarrhea incidence or prevalence*					
5	2 cRCT, 3 QE	3 very low, 1 low, 1 moderate quality study	I^2^ = 81% Studies favoured intervention, control, or showed no effect	Children 0–5 years; low and middle income countries (Afghanistan, Bangladesh, Pakistan, Panama, Uzbekistan)	Very low (considerable heterogeneity, non-significant pooled estimate)
Point-Of-Use Water Treatment Interventions					
*Intervention: Water filters and water disinfection, Outcome: Diarrhea incidence or prevalence*					
32	15 RCT, 12 cRCT, 5 QE	17 very low, 11 low, 4 moderate quality studies	I^2^ = 89% Studies either favoured intervention or showed no effect	Children 0–5 years; low and middle income countries (Afghanistan, Bangladesh, Bolivia, Brazil, Cambodia, Colombia, Democratic Republic of Congo, Dominican Republic, Ethiopia, Ghana, Guatemala, Haiti, India, Honduras, Kenya, Pakistan, Saudi Arabia [rural], South Africa, Uzbekistan, Zimbabwe)	Low (15 studies were low or moderate quality, large significant magnitude of effect, considerable heterogeneity warrants further research on the magnitude of the benefit)
*Intervention: Water filters, Outcome: Diarrhea incidence or prevalence*					
13	8 RCT, 4 cRCT, 1 QE	8 very low, 5 low quality studies	I^2^ = 84% Studies generally favoured intervention	Children 0–5 years; low and middle income countries (Bolivia, Cambodia, Colombia, Democratic Republic of Congo, Dominican Republic, Ethiopia, Ghana, Honduras, Kenya, South Africa, Zimbabwe)	Very low (mostly very low quality studies)
*Intervention: Water disinfection, Outcome: Diarrhea incidence or prevalence*					
19	7 RCT, 8 cRCT, 4 QE	9 very low, 6 low, 4 moderate quality studies	I^2^ = 87% Studies either favoured intervention or showed no effect	Children 0–5 years; low and middle income countries (Afghanistan, Bangladesh, Bolivia, Brazil, Cambodia, India, Ethiopia, Ghana, Guatemala, Haiti, Kenya, Pakistan, Saudi Arabia [rural], Uzbekistan)	Low (studies ranged from very low to moderate quality, large significant magnitude of effect, considerable heterogeneity warrants further research on the magnitude of the benefit)
Hand Washing Education with Soap Interventions					
*Outcome: Diarrhea incidence or prevalence*					
6	4 cRCT, 2 QE	5 very low, 1 low quality study	I^2^ = 81% Studies either favoured intervention or showed no effect	Children 0–5 years; low and middle income countries (Bangladesh, India, Myanmar, Nepal, Pakistan)	Very low (most studies very low quality, considerable heterogeneity)

### Water quality improvement

**Fig. 2:**
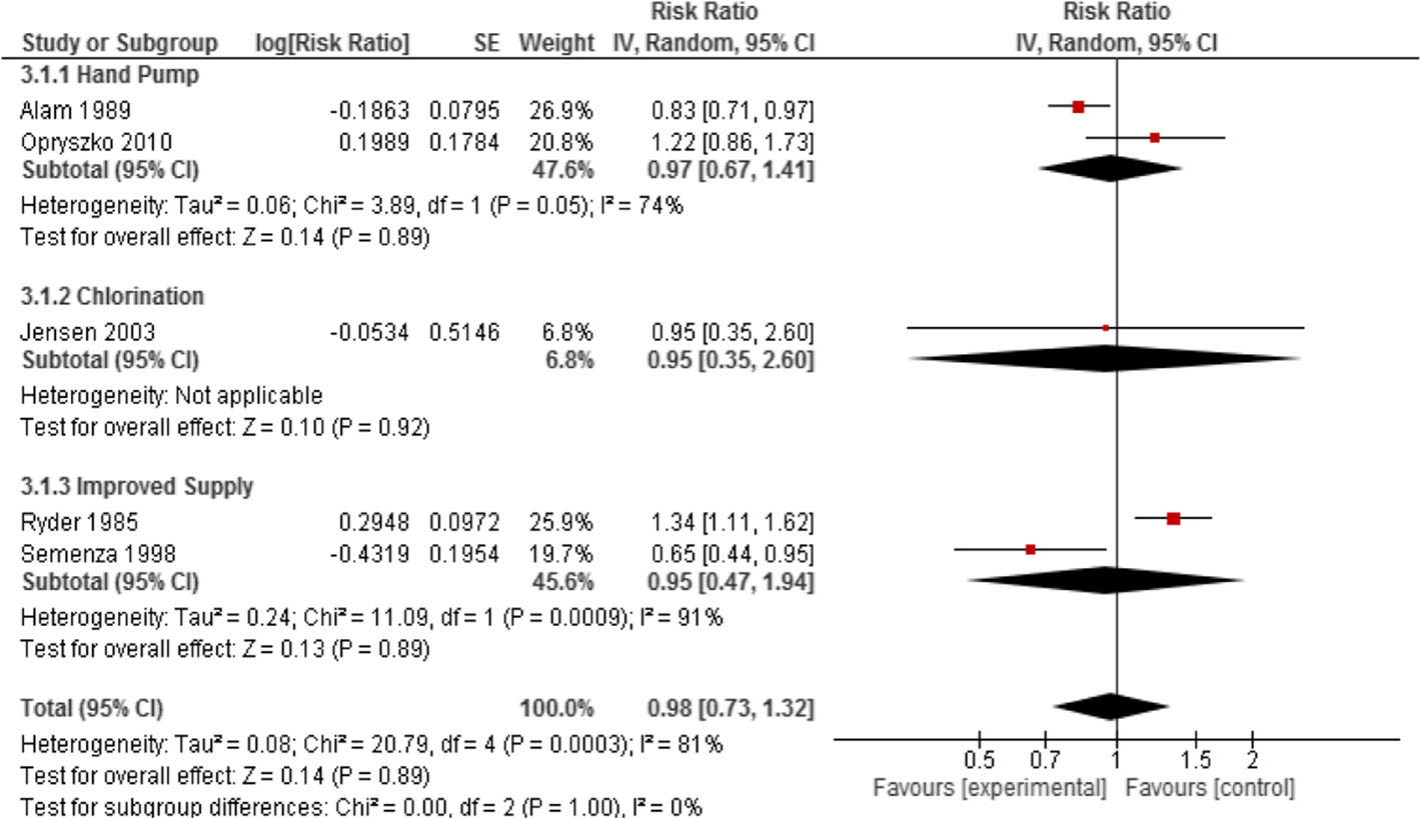
Forest plot for the effect of water quality improvement at source on diarrhea

We identified five studies that provided water quality improvement intervention at the water supply [17–21]; two studies were cRCTs and three were QE. All of these studies were conducted in low and middle-income (LMIC) settings and the interventions included improved supply systems, hand pumps, and water disinfection (chlorination). The combined analysis suggested no effect of water quality interventions at source on risk of diarrhea (pooled RR: 0.98 95%CI: 0.73, 1.32) and the subgroup analyses for the various interventions also suggested no effects (Fig. 2).

**Fig. 3:**
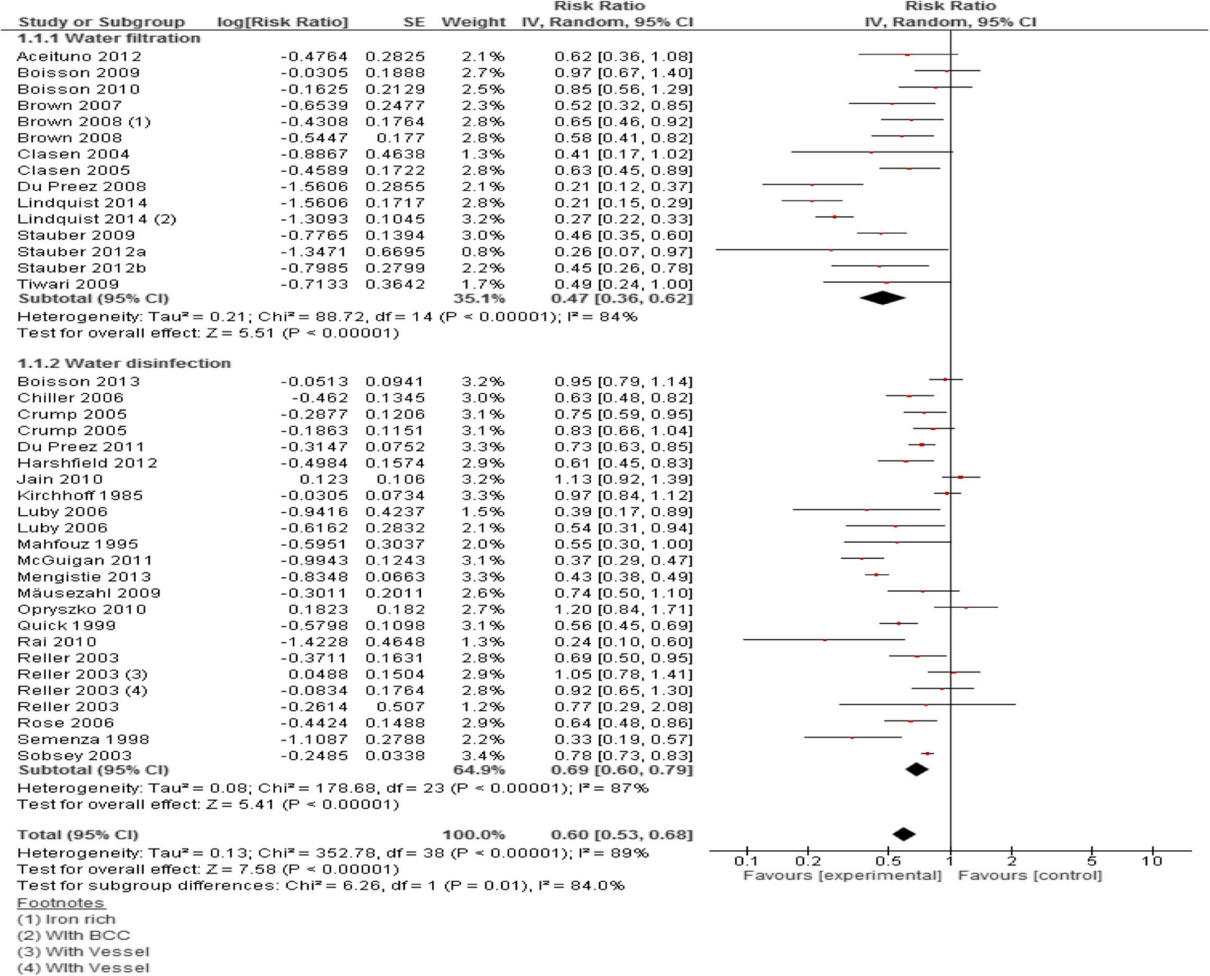
Forest plot for the effect of water quality improvement at point-of-use on diarrhea

**Fig. 4:**
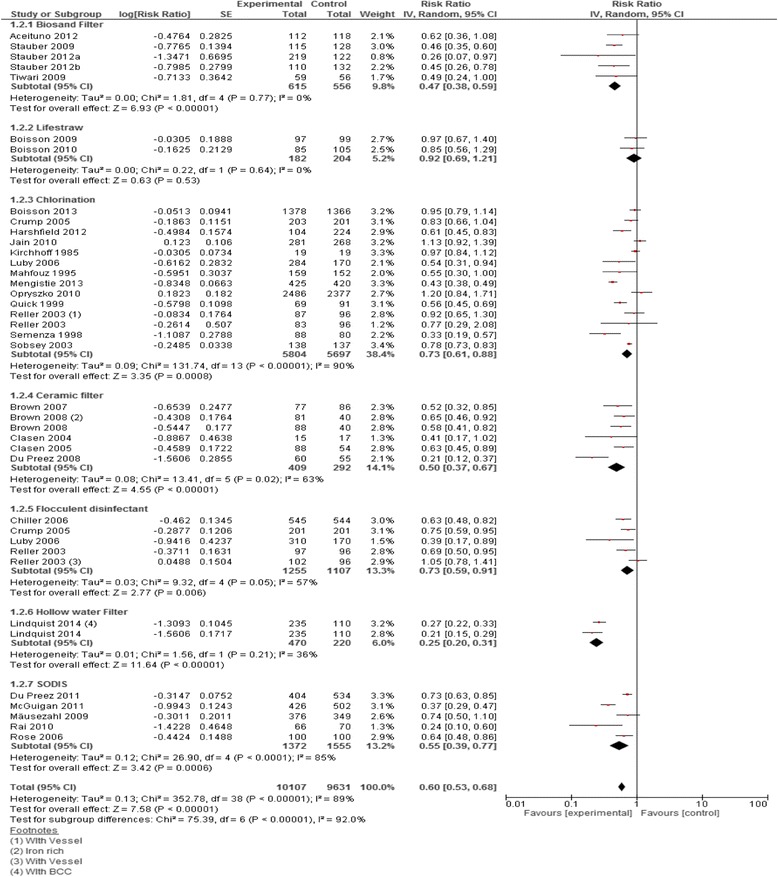
Forest plot for the effect of water quality improvement at point-of-use on diarrhea

We identified 32 studies for inclusion in analysis that had a water quality improvement intervention at point-of-use [18, 21–51]; 27 of these were RCTs or cRCTs while five were QE study designs. Studies were from Africa (Kenya, Ghana, Democratic Republic of the Congo, Ethiopia, Zimbabwe, South Africa), Asia (Bangladesh, Pakistan, India, Afghanistan, Saudi Arabia, Uzbekistan, Cambodia), South America (Bolivia, Brazil, Colombia), Central America (Honduras, Guatemala), and the Caribbean (Haiti, Dominican Republic). There were a range of interventions delivered which were broadly categorized into ‘water filtration’ [22–34] and ‘water disinfection’ [18, 21, 35–51] interventions. Water filtration interventions included biosand filters, ceramic filters, lifestraws, and hollow water filters while disinfection interventions included chlorination, use of flocculent-disinfectant, and solar disinfection (SODIS). One study reported the impact of flocculent-disinfectant on all-cause mortality in children under the age of two years and reported a 65% reduction (RR: 0.35, 95%CI: 0.13, 0.94) [37]. Overall, ‘water quality interventions at the point-of-use’ showed a significant decrease in risk of diarrhea by 40% (RR: 0.60, 95%CI: 0.53, 0.68), while the subgroup analyses suggested a 53% decrease (pooled RR: 0.47, 95% CI: 0.36, 0.62) with respect to water filtration and a 31% decrease (pooled RR: 0.69, 95% CI: 0.60, 0.79) with respect to water disinfection (Fig. 3). A further subgroup analysis suggested a significant effect for each of the specific interventions except for lifestraw (Fig. 4).

### Handwashing with soap

**Fig. 5:**
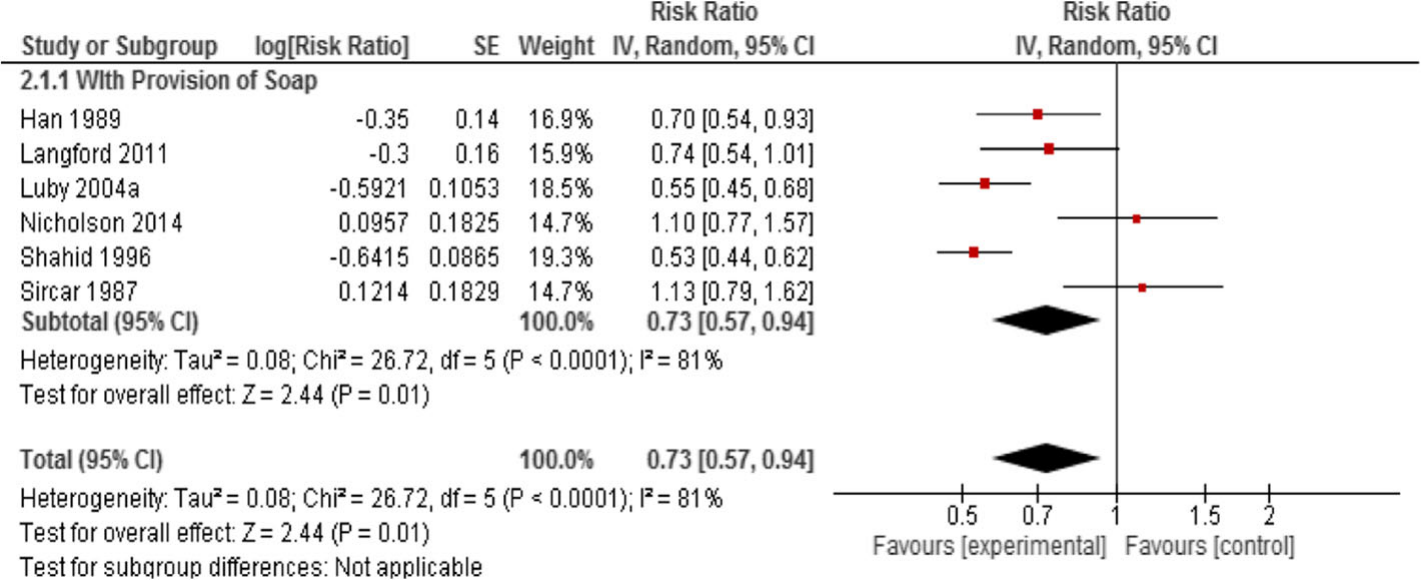
Forest plot for the effect of handwashing with soap on diarrhea

We identified six studies which evaluated the effect of handwashing with soap [52–57]; four were cRCTs and two were QE study designs. All studies were conducted in South Asian countries. Study participants were provided soap with education about handwashing before eating or food handling, after defecation or handling of child stools, or a combination of these. No study reported on mortality and the analysis suggests that handwashing with soap leads to a 27% decrease in risk of diarrhea (pooled RR: 0.73, 95% CI: 0.57, 0.94) (Fig. 5).

### Excreta disposal

The search for studies for excreta disposal interventions resulted in few studies with study designs that met our inclusion criteria, and some studies had other interventions including water supply interventions or multiple interventions evaluated together, hence the impact of excreta disposal alone could not be ascertained [58–63]. One study was included which showed that latrine construction in India increased mean village-level latrine coverage from 9% of households to 63% in the intervention group, but there was no impact on the risk of diarrhea in children younger than 5 years (RR: 0.97, 95% CI: 0.83–1.12)[64].

## Discussion

The review findings suggest that point-of-use water quality improvement interventions are effective in reducing the risk of diarrhea by 40% in children 0–5 years old in communities living in LMICs and subgroup analyses suggest greater impacts with water filtration (53%) than with water disinfection (31%). In addition, hand washing promotion with soap can lead to 27% reduction in risk of diarrhea. Evidence for the effect of water supply interventions at source and safe excreta of stools is insufficient to conclude an impact on childhood diarrhea. The overall quality of evidence is low to very low since most studies were not blinded – a design which may be difficult and unethical to adhere to in this context.

We did a de novo search for studies with specific inclusion and exclusion criteria which could provide precise estimates for inclusion in LiST, and also updated the evidence since the last LiST review which was published more than five years ago. As only one study for water quality improvement assessed all-cause mortality and the number of events were less than 50 [37], we propose our estimates based on diarrhea risk reductions 40% and 27% for point-of-use water quality interventions and handwashing with soap respectively. The evidence for water quality interventions at source and safe excreta disposal is too limited to propose an estimate for LiST.

Our results are broadly consistent with prior reviews in this area [3–10], though the estimated magnitudes of intervention effect are different than those proposed by Cairncross et al. [7], which were 17%% and 48% for water quality interventions and handwashing with soap, respectively. In addition to the inclusion of more recent evidence in the present review, the differences between the present and previous LiST review may be attributable to choice of effect measure, study designs, populations and settings. The previous LiST review [7] included observational studies and evidence from settings other than those in LMIC communities, including studies conducted in schools, daycare centres, refugee camps, out-patient clinics, and hospitals, and it also included studies conducted in children over the age of five. The previous review also included studies with primary outcomes of typhoid, cholera or dysentery, while we only included studies reporting on diarrhea. We propose an estimate for water quality improvement at point-of-use only, as the evidence is more consistent, while there is limited evidence for water quality improvement at source and suggest a non-significant impact on diarrhea.

While point-of-use water quality interventions and handwashing promotion with soap appear to be effective in reducing diarrhea, much of the evidence is from trials conducted in small populations over short time periods. More evidence is needed on compliance over a longer duration to assess sustainability. The challenge is to find ways of encouraging people to maintain handwashing habits in the longer term. The need to conduct research with longer follow-up duration using a structured method of assessing the primary outcome is pertinent, since it has been observed that the choice of method may have significant effects on the precision of estimates. Outcome assessors should be blinded so as to reduce the bias in estimates of effect size. Self-reported outcome measurements such as diarrhea frequency are prone to recall and other biases, which contributed in part to the low methodological quality ratings overall. There are a number of large scale trials underway with results eagerly awaited which might shed further light on the short and long-term impact of WASH interventions at scale [65].

The importance of WASH strategies for reducing childhood diarrhea is fairly established, but the challenge remains to make their availability universal. Sustainable Development Goal (SDG) 6 covers the whole water cycle, and includes targets for universal access to drinking water, sanitation, and hygiene that are significantly more ambitious than the previous targets of the Millennium Development Goals (MDGs). To accomplish these goals, changing behaviours and social norms is essential, governance and accountability should be ensured, and inequalities will have to be eliminated.

## Abbreviations

CHERG: Child Health Epidemiology Reference Group
cRCT: cluster randomized controlled trials
GRADE: Grading of Recommendations Assessment, Development and Evaluation
LMIC: low and middle-income countries
QE: quasi-experimental
RCT: randomized controlled trials
SDG: Sustainable Development Goals
WASH: water, sanitation and hygiene
